# Barriers and facilitators to implementing Advanced HIV Disease screening at secondary referral hospital -Malawi: Asequential exploratory mixed method-study

**DOI:** 10.21203/rs.3.rs-2602019/v1

**Published:** 2023-03-15

**Authors:** Brany Mithi, Agatha Bula, Lester Kapanda, Fatsani Ngwalangwa, Evanson Z Sambala

**Affiliations:** Kamuzu University of Health Sciences (KUHeS), School of Community Health and environmental health; University of North Carolina (UNC) Project; Elizabeth Glaser Pediatric AIDS Foundation (EGPAF); Kamuzu University of Health Sciences (KUHeS), School of Community Health and environmental health; Kamuzu University of Health Sciences (KUHeS), School of Community Health and environmental health

**Keywords:** ART, Consolidated framework of implementation research (CFIR), People Living with HIV, Advanced HIV Disease (AHD)

## Abstract

**Background:**

Malawi continues to register increased HIV/AIDs mortality despite increased expansion of ART services. One of the strategies for reducing AIDS related deaths outlined in the Malawi National HIV Strategic Plan (NSP) is scaling up screening for AHD in all antiretroviral therapy (ART) screening sites. This study investigated factors influencing the implementation of the advanced HIV disease (AHD) screening package at Rumphi District Hospital, Malawi.

**Methods:**

We conducted a mixed method, sequential exploratory study from March, 2022 to July, 2022. The study was guided by a consolidated framework of implementation research (CFIR). Interviews were administered to key healthcare providers, purposively selected from various hospital departments. Transcripts were organized and coded using NVivo 12 software with thematically predefined CFIR constructs. Newly HIV-positive client records extracted from ART cards (July –Dec, 2021) were analyzed using STATA 14 which generated table of proportions, means and standard deviations.

**Results:**

Out of 101 data records of the new ART clients reviewed, 60% {(n = 61) had no documented results for CD4 Cell count as a baseline screening test for AHD. Four major themes emerged as barriers: complexity of the intervention, poor work coordination, limited resources to support the expansion of point of care services for AHD, knowledge and information gap among providers. Technical support from MoH implementing partners and the availability of committed focal leaders coordinating HIV programs emerged as major facilitators of AHD screening package.

**Conclusion:**

The study has identified major contextual barriers to AHD screening affecting work coordination and client linkage to care. Improving coverage of AHD screening services would therefore require overcoming the existing barriers such communication and information gaps.

## Background

Despite the significant progress in expanding access to antiretroviral therapy (ART) in Malawi, AIDS-related deaths plateaued over the recent years [1, 2]. HIV Epidemiological Estimates for Malawi indicate that 10 800 deaths were registered among 987 000 adults (15 + years) living with HIV in 2020 [3]. The prevailing HIV mortality rates have been attributed to opportunistic infections (OIs) such as Tuberculosis (TB), and Cryptococcol meningitis often associate with Advanced HIV Disease (AHD) [4]. The impact of these opportunistic infectious diseases has been immense on the performance of healthcare systems in poorly resourced settings particularly in sub-Saharan Africa [5, 6].

While Malawi has made gains in the UNAIDS 95-95-95 treatment and viral load suppression targets (88.3%, 97.9%, and 96.9%), it is yet to reach the 95% target for HIV positive individuals who know their status [1]. The majority of HIV positive individuals unaware of their status often present with AHD due to late HIV diagnosis which increases the risk of morbidity, mortality as well as HIV transmission [7, 8]. Therefore, identifying newly diagnosed HIV clients in advanced HIV stage through CD4 Cell count testing and linking them to care, provide a means for preventing morbidity and mortality among people living with HIV (PLWH) initiating ART [9].

One of the strategies for reducing AIDS mortality outlined in the Malawi National HIV Strategic Plan (NSP) is scaling up screening for AHD in all antiretroviral therapy (ART) screening sites [10]. This strategy compliments the 2017 WHO-recommended AHD package of care integrated in the Malawi HIV Clinical Management guideline [11]. WHO defines advanced HIV disease as CD4 Cell count of less than 200 cells/μL or HIV clinical stages 3 and 4 [9]. Since the roll- out of the AHD screening package in 2020 in Malawi, several people living with HIV have been diagnosed with opportunistic infections and linked to care, owing to the use of point of care rapid devices such as Urine LAM, Cryptococcol antigen (CrAg) and PIMA machine for CD4 cell count. Subsequently, AIDS mortality rate due to OIs de-masked by immune-reconstitution inflammatory syndrome (IRIS) often common in the first month of ART initiation has gone down [8, 9]. Considering the high prevalence of TB and Cryptococcal meningitis among clients with AHD, there is need to scale up AHD screening as an entry point to care for newly diagnosed HIV positive clients of which majority are asymptomatic[4].

However, the implementation of AHD screening in various healthcare facilities has not been smooth. The HIV coverage quality and impact network (CQUIN) project in Malawi singled out COVID-19 as the major obstacle to delivery of AHD package of care as it led to decreased in the number of PLHIV attendance and delays in AHD trainings for healthcare providers [2]. Studies conducted in tertiary referral hospitals have also established barriers at the system and healthcare providers’ level such as limited trained personnel, and inconsistencies in testing as key challenges in implementation of AHD diagnostic tests that often leads to non-compliance to AHD guidelines and poor identification of AHD cases [8, 10, 11].

Although Ministry of Health (MoH)’s response has focused on conducting mentorship and onsite supervisions as well as expanding screening sites, without understanding the nature and root cause of the existing contextual barriers, interventions are likely to be ineffective. Currently, local data about contextual barriers from secondary referral facilities is missing. Most previous AHD studies focused on hospitalized patients in tertiary referral hospitals. Our study, therefore, sought to investigate factors influencing implementation of AHD screening package among newly HIV-diagnosed clients in one of the secondary referral facilities in Malawi. The findings will provide a framework for designing strategies for overcoming existing implementation barriers.

**Figure 1:**
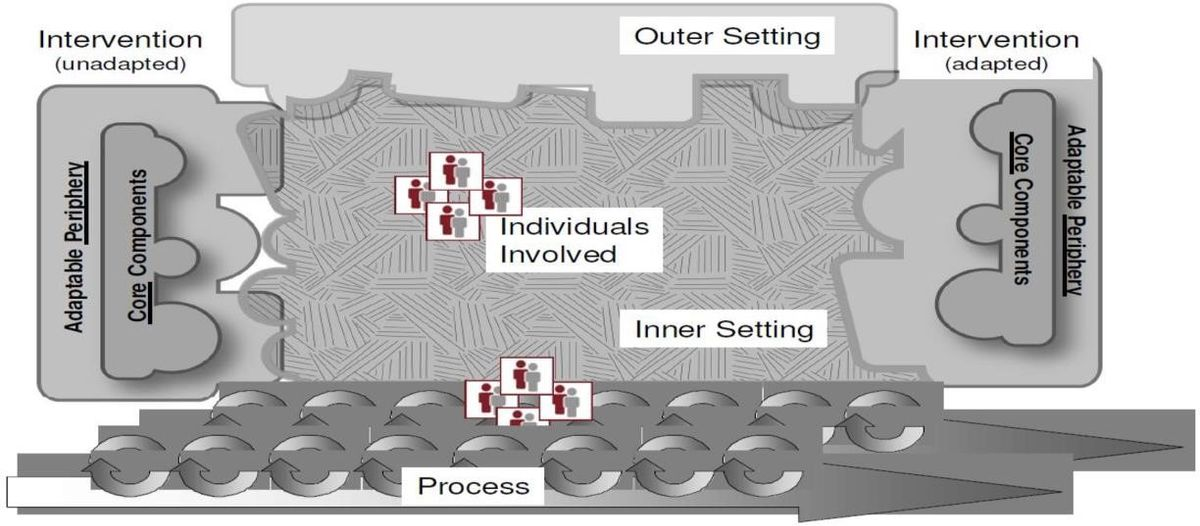
Major five domains of the CFIR and their relationships

In our study, we applied the Consolidated Framework for Implementation Research (CFIR) as an analytical framework to explore contextual barriers and facilitators from multiple levels within the healthcare system [16]. The CFIR was selected because it provides a comprehensive understanding of the contextual determinants of health service innovations of proven effectiveness [11, 12]. This conceptual framework comprises of five major domains; intervention characteristic, outer setting, inner setting, characteristic of individuals, and the process. These domains have a total of 39 constructs that ensure maximum exploration of contextual factors influencing the implementation of an intervention. CFIR has been used in a number of interventional studies and programmes ranging from communicable and non-communicable diseases, to nutrition and others[14, 15, 16, 17]. The Fig. 1 shows a Consolidated Framework for Implementation Research, adapted from Yuan Lu, 2018 [23].

## Methods

### Study design and site

**Figure 2:**
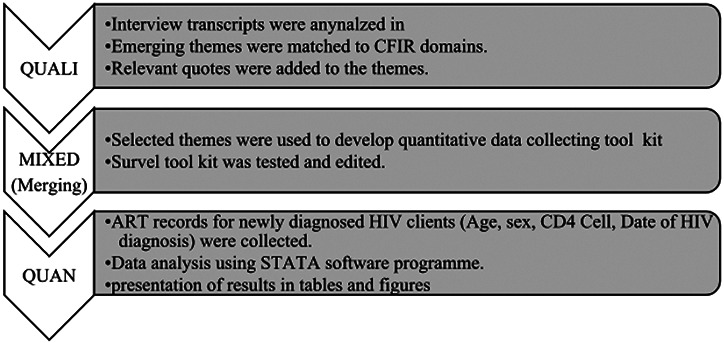
Mixed method data integration process

We conducted an exploratory mixed methods study to explore the contextual factors and gain a more comprehensive understanding of the impact of the barriers to delivery of AHD screening services. Data was collected sequentially in two phases: the first phase involved collection and analysis of qualitative data. This was followed by the building approach where themes and participant quotes of preliminary qualitative findings informed data collection procedure for secondary quantitative variables. Figure 2 describe the process of mixed method data merging and reporting.

The study was conducted at Rumphi District Hospital, Northern Malawi. The district covers an area of 4,769 km.^2^, with a population of 128 360 people. At the time of data collection, the hospital ART Clinic supported by Lighthouse organization, had 8 659 clients on ART The facility was chosen because it implements the AHD management package of care and faces various implementation barriers including shortage of AHD trained providers and the impact of COVID 19 pandemic according to the MoH department of HIV/AIDS CQUIN report [2]. AHD screening was collaboratively done by laboratory staff, clinicians and nurses with all the three AHD point of care tests (CD4 Cell count, serum CrAg and Urine LAM) conducted in the main laboratory.

### Study population and sample size

We purposively selected a homogenous sample of 10 health care workers including laboratory staff, nurses and clinicians who were actively involved in the implementation of the AHD management package of care. In-charges were tasked to identify key informants through snowballing. The addition of quantitative data with 101 ART reviewed records of newly diagnosed HIV positive clients covering two quarterly months (July to Dec, 2021) helped to further validate themes from qualitative data analyses.

### Qualitative data collection

Using the CFIR selected constructs, we designed an interview guide which was pre-tested during demonstration interviews between research assistants and four healthcare workers at the hospital to determine the flow and appropriateness of the questions. All interviews were conducted in English but participants were allowed to express themselves in Chichewa, the local language. Each IDI lasted for about 30–40 minutes. During the interview process, field notes were written, highlighting important points and any new information emerging. All IDIs in local language were audio recorded, transcribed and translated into English.

Data for newly HIV-diagnosed client was collected with details such as patient name, age, date of HIV diagnosis and whether the client was checked for CD4 Cell count or not. The final list was de-identified and assigned numbers before transferring it into an excel sheet, ready for analysis.

### Qualitative data analysis

We first analyzed qualitative data soon after the IDI using deductive method where data was coded according to the CFIR framework of analysis, with themes categorized based on defined barriers or facilitators. A thematic analysis was employed in order to find patterns across the interview data in relation to our research questions. During the initial phase of analysis, BM and AG reviewed the interview transcripts for accuracy. Following familiarization with data, a codebook was developed based CFIR domains and most applicable constructs to guide coding and categorization of data using NVivo software version 12 (QSR International). The codebook which had clearly defined parent and child nodes was continuously reviewed and edited by authors. During the analysis, transcripts were entered into NVivo where codes were indexed to sections of the transcripts. Coded text categories were further examined for patterns. Finally, data was abstracted and interpreted, with direct quotations of the some respondents’ opinions included in the final report under various themes.

### Quantitative data analysis

ART data records in the excel sheet was imported into STATA 14.0 software programme and run to generate descriptive statistical analyses in form of proportions, mean, range and standard deviation. Findings were presented in form of tables and figures.

### Ethical Consideration

The study was approved by the College of Medicine Research and Ethics Committee (COMREC), certificate number P.01/22/3542. Consent process was followed with each of the interviewees and all interviews were conducted at a place convenient to the participants. Both qualitative and quantitative data with personal information was de-identified.

## Results

### Demographic characteristic of participants

Table 1 summarizes the total number and background characteristics of participants involved in the study. Out of the 10 IDIs, four (40%) were clinicians, three (30%) laboratory scientists, and the other three (30%) were nurses. Most of the participants were males (*n* = 8, 80%). Participants age range was 27 to 59 years old with a mean age of 43.3 (± 8.2). These participants have worked in hospital between 10 to 25 years (Mean = 15.5, ± 5.0). 50% (n = 5) were degree holders of various health related qualifications, while about 40% (n = 4) had diplomas. The majority of the participants attended either a formal AHD training or an on-job training and were working in departments which are actively involved in AHD screening.

Table 2 displays the background characteristics of the newly HIV diagnosed clients from ART data records of July to December, 2021. There were 101 study participants with a balance distribution of sex (Male = 49.5%, Female = 50.5%) and mean age of 35years.

**Figure 3:**
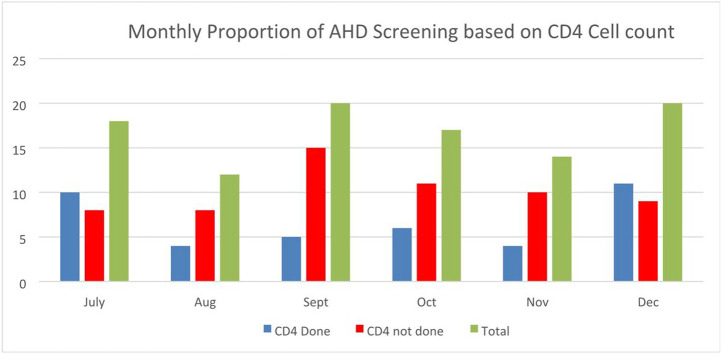
Clients screened for AHD using CD4 Cell count

All major qualitative themes indicated that healthcare level barriers significantly contributed to poor linkage to care among eligible new ART clients. Respondents from ART clinic highlighted poor communication structures, distance and congestion at the laboratory as factors that led to clients long waiting time and loss to follow ups. Thus, the subsequent analysis of secondary data of ART client records provided evidence of the impact of the existing barriers on client access to AHD screening. For instance, out of the 101 ART records reviewed, 60% {(n = 61) of the newly HIV-diagnosed clients had no documented results for AHD with CD4 cell count as a baseline test. Overall, both the third and fourth quarterly months had fewer clients screened for AHD {3rd Q, 38% [19/50]; 4th Q, 41% [21/51]}. As shown in Fig. 3, low AHD screening rate was registered for four consecutive months of August 33% (4/12), September 25% (5/20), October 35% (6/17) and November 30% (4/14).

### Contextual barriers and facilitators of AHD screening implementation

Due to overlapping of CFIR domains and constructs, our findings on facilitators have been presented in four major themes as informed by CFIR framework; 1). Complexity of the intervention, 2). Limited supporting resources, 3). Poor work coordination, 4). Knowledge gap. Enablers of implementation have been presented thematically as external and internal facilitators. More barriers and facilitators has been summarized in the Table 3 and Table 4 respectively.

### Complexity of the intervention

The implementation process was found to be affected by factors relating to the complexity of AHD screening. Providers reported the implementation process is quite involving and cumbersome with the inclusion of various departments and multiple lab tests which also increases patients waiting time. Any new HIV positive client has to undergo rapid screening tests for TB-LAM and CrAg if CD4 count was less than 200 cells/μL, before finally linked to treatment. Moreover, results recording was reported to be a challenge owing to a substandard register for the three AHD rapid tests. One of the respondents from the laboratory commented: *“We have been given a CD4 register from the MoH, but it is missing these other tests. So if you are documenting these tests results, you will be using a separate improvised register which is cumbersome.”* **[Lab tech].** This result in missing data as other technicians are reportedly not documenting results.

### Limited supporting resources

Respondents acknowledged the absence of supporting resources such as posters and testing algorithm which contributed to poor execution of AHD screening by the majority providers who never attended any formal training. This was classified as a potential barrier from the inner setting. One respondent from ART clinic said: *“The government has delayed to provide the posters because we should have pasted some in the wards for our friends who have less knowledge to understand what’s going on”* **[ART Clinician].**

Most respondents argued that it was difficult for other departments to start conducting the AHD diagnostic tests outside laboratory without necessary laboratory equipment such as centrifuge, pipettes, and waste management facilities. Responding to the question as what could be the way forward, a DHMT member said: *“the laboratory should continue providing the POCT services up until the facility has enough resources to extend it to ART clinic”* **[DHMT rep].**

As a way of preparing the ART clinic for POCT testing and to ensure services are not interrupted due to staff engagements, a representative from Lighthouse suggested that ‘lay cadres be trained in conducting rapid tests to prevent service interruption due to staff engagements. *“In other sites they trained the HDAs and ART Clerks so it becomes easy when all nurses and clinicians are not available”* **[LH Clinician]**

### Poor work coordination

Physical and technological barriers in the inner setting affected work coordination among teams involved in the execution of the intervention. Respondents raised concerns about the distance between ART clinic and the laboratory which contributed to poor tracking of new ART clients. They proposed relocation of POCT services to ART clinic as a solution to client loss to follow up due to distance and congestion at the laboratory. One provider made reference to the policy that require AHD testing services to be conducted at the ART Clinic, arguing that the current arrangement defeated the intended purpose of providing quick services to the eligible clients. *“If the patient has to go to the lab to access testing services rather than within the ART clinic then it’s no longer point of care.”* **[LH Nurse].**

Absence of such communication systems compromised formal and informal information sharing practices which had a negative impact on work coordination among different working teams. Without ground telephone system in place, implementers used personal gadgets for communication and often walked on foot to and from laboratory whenever they run short of airtime to inquire about testing services. *“Sometimes there are stockouts of some reagents in the laboratory. It’s very hard to know until you send a client and comes back without a test”* **[ART Nurse]**.

### Knowledge gap

Respondents emphasized that knowledge gap among healthcare providers significantly contributed to poor adherence to guidelines as well as under-screening of AHD among newly HIV-diagnosed clients. Untrained providers were said to have decreased self-efficacy to conduct AHD screening. One of the participants argued that trained AHD providers were not adequate such that some departments lacked trained healthcare personnel: *“In the male ward department, I think am the only one trained which means if am not available then we have problems***[Clinician Mw**].

### External facilitators

Besides the availability of MoH policy documents such as guidelines and SOPs supporting implementation of AHD screening, the facility was found to be well supported by MoH and PEPFAR implementing partners. For instance, Lighthouse organization provided technical support in form of personnel and data collecting tools, in addition to facilitation of integrated HIV programme review meetings at district level. On the other hand, MoH facilitated integrated HIV mentorships and supervision programmes as well as bi-annual AHD review meetings where teams from different district hospitals come together and learn from each other. “ *This week a team from Department of HIV/AIDs has been going around in different health facilities carrying out mentorship programmes”***[MA]**

### Internal facilitators

Additionally, respondents mentioned that the availability of two AHD TOT trained implementation leads who were also coordinators for HIV Testing Services and ART helped to facilitate the implementation of the AHD screening package. One of the coordinators further explained that their primary responsibility was to ensure that AHD providers comply with the available guidelines: *“So as a trainer and Coordinator I look to it that the samples are collected adequately and run according to the specified AHD guidelines…The ART coordinator makes sure that that all who have tested HIV positive for the first time undergo CD4 test as the baseline test”***[focal].** The implementation leads demonstrated in-depth knowledge of AHD management package and the zeal to support the implementation efforts.

## Discussion

In this mixed methods study, most of the contextual barriers were interlinked and cross-cutting among CFIR domains, with the majority emerging from the inner setting. Major implementation barriers in the inner setting include unavailable communication systems and information gap as well as inadequate resources to support expansion of POCT to other screening sites. Merging and integration of the qualitative and quantitative findings, showed evidence of impact of barriers as expressed in qualitative themes and the coverage expressed as proportions in the surveyed ART clients.

Most respondents reported poor AHD screening coordination among implementers due to communication challenges owing to the absence of ground telephone system and other social platforms for communicating and sharing relevant AHD updates. Communication system barriers existing between healthcare providers and their clients have also been documented elsewhere [24]. Thus, the use of social mobile platforms (SMPs) with instant messaging applications such as WhatsApp is highly recommended. In Ghana, WhatsApp platforms successfully facilitated networking and communication among healthcare workers which offered collaborative opportunities for TB screening and case detection [15]. SMPs provide a fast and affordable means of information sharing between healthcare providers in the management of clients [20, 21, 22].

Some respondents also suggested quarterly cross departmental meetings to facilitate information sharing of AHD updates among implementers. AHD services could also be integrated into ART and TB programmes which are well established and sufficiently supported by MoH PEPFAR implementing partners. Integration of related health programs reduces duplication of services, is cost-effective, and as well as efficient [23, 24].

Facility work flow and distance were also identified as potential barriers as they led to long AHD screening pathways for clients. Similar findings were also reported in a study conducted in the Eastern Africa where overcrowding, long waiting times and lack of resources emerged as prominent barriers [30]. In our study, we noted long client waiting time primarily due to the fact that testing was conducted only in a medical laboratory which also performed other testing services for in-patients and out-patients. Most respondents wished the Point of Care Testing (POCT) for AHD diagnostic services were stationed at ART clinic where the majority of new ART clients report for care. This was underlined as a solution to the problem of long waiting time and loss to follow-up due to congestion at the main laboratory.

However, financial and diagnostic resources are required for the establishment of a functional compartment for AHD services at ART clinic. A recent study at a tertiary healthcare facility in Malawi estimated a capital investment cost of establishing and equipping an advanced HIV disease room for diagnostic tests to be U$10,708 [12]. This amount is affordable for MoH with the support from the U.S. President’s Emergency Plan for AIDs Relief (PEPFAR) which has recently focused on decreasing mortality among PLWH by addressing advanced HIV Disease and its associated opportunistic infections [31].

As a means to increase access to knowledge and information about AHD screening, formal AHD trainings are highly recommended. In our study, stakeholders alluded that healthcare providers with less knowledge of the intervention lacked confidence and self-efficacy when conducting AHD screening in various screening points within the hospital. This led to low screening coverage as many eligible clients were missed out. Such findings corresponds with programme evaluation reports which highlights inadequately trained providers as a stumbling block to a successful implementation of the HIV programmes [27, 28, 29, 30]. Limited trained personnel is a common challenge in the delivery of AHD screening and diagnostic services [14]. Therefore, improving knowledge and information through both formal and informal training is therefore paramount [36]. There is also urgent need for hospital implementation leads to make use of learning platforms such as morning handover reporting and weekly ground ward rounds which we found not to be adequately utilized by nurses and clinicians.

Our investigation also revealed that posters for AHD screening were not available in all screening points. Nursing and clinical staff with less knowledge of the intervention had no access to such reference material. This widened the knowledge and information gap and partly contributed to non-compliance of AHD screening guidelines. Posters with information about eligibility criteria and testing algorithm could be a significant step towards improving adherence to guidelines by health care providers. One advantage of using posters is that they are readily available, hence provides instant knowledge and awareness of the intervention [37].

Most trained healthcare workers perceive the AHD screening package of care to have more clinical benefits to their clients. This is a facilitator of the AHD screening as health providers are motivated by positive clinical outcomes of the intervention. Many scientific papers have reported various benefits of the WHO AHD management package of care which includes improvement in diagnosis and management of opportunistic conditions, leading to improvement of quality of life for PLWH who would otherwise die to HIV related complications [2, 9, 30, 34, 35].

Supervision also tops as strong facilitators of implementation of interventional programmes in HIV/TB [40]. Similar findings emerged in our study in which external networks and partnerships with other health organizations significantly contributed to efficient delivery of the AHD screening services through capacity building. With MoH led mentorship and supervision programmes, major implementation gaps were identified and quickly addressed. Besides, Lighthouse organization technical team was very key in supporting AHD package through facilitation of AHD trainings, review meetings and involvement in reporting monthly data for evaluation.

A successful implementation of an intervention also require presence of experienced focal leaders to oversee the implementation process [41]. Our study site had two focal persons: one with a laboratory background coordinating HIV Testing Services (HTS), while the other one with a clinical background, coordinated ART services. Their extensive knowledge of the AHD management package of care, and high level of commitment, facilitated the implementation of this complex intervention. Globally, there is a tremendous need for well-trained leaders in both healthcare and advocacy, especially in countries with high HIV prevalence and limited human resource capacity [38, 39]. This enables smooth implementation of interventional programmes aiming at reducing the burden of HIV infections and mortality.

### Study limitations

We acknowledge that the study wasn’t done on large scale, involving multiple health facilities implementing the WHO recommended AHD package of care due to time and financial constraints.

We also wished we could have interviewed clients who underwent AHD screening services to better understand their experiences. One of the strength of this study is that we employed mixed methods, of which qualitative and quantitative data triangulation improved validity of the findings.

## Conclusion

The study has identified major contextual barriers to AHD screening, affecting work coordination and client linkage to care. The study findings provide an insight of the existing contextual barriers as well as comprehensive approaches for HIV programme implementers to design barrier-tailored strategies for optimizing AHD screening services in secondary healthcare facilities. Improving access and coverage of AHD screening services would therefore require overcoming the existing barriers in the facility set-up. There is also need to maintain good institutional partnerships with MoH and other external organizations to ensure continued technical support towards AHD screening. Use of CFIR framework of analysis and mixed methods study designs in evaluation of

## Figures and Tables

**Table 1:** Demographic characteristic of participants

Sex		Age		Years of service		Profession		Level of Education	
**Male**	08 (80%)	Mean	43.3	Mean	15.9	Clinicians	4 [40%]	Masters	0 [0%]
**Female**	02 (20%)	SD	±8.2	SD	±5.0	Lab scientists	3 [30%]	Degree	5 [50%]
		Min	30	Min	10	Nurses	3 [30%]	Diploma	4 [40%]
		Max	57	Max	25			Certificate	1 [10%]

**Table 2:** background characteristic of ART clients

Proportion CD4status				
Number of Obs	Mean	Std. Dev	Min	Max
**Age**		**35.14851**	**11.85781**	**70**
Proportion estimation				
	Proportion Std. Err. [95% Conf. Interval]			
CD4 Status				
.3960396	.0489073		.3041413	.4959164
.6039604	.0489073		.5040836	.6958587

**Table 3:** Summary of contextual barrier

CFIR Constructs	Barrier	Strategy
1. Networks and communications	Absence of ground telephone system.	Installation of ground phone system
	No regular meetings for implementers	Cross departmental meetings. Use of WhatsApp group forums
2. Information technology infrastructure	Paper based technology is slow and cumbersome.	Extension of LIMS to wards for quick accessibility of results
3. Physical structure	Distance between ART and Lab leads to clients Loss to follow up and poor linkage to care.	Establish ART POCT for AHD
4. Complexity	Multiple steps and departments involved in the implementation process	Establish ART POCT for AHD
	Poor results documentation.	Need for a consolidated standard register for easy results documentation & retrieval.
	Multiple registers for different tests.	
5. Available resources	Lack of financial support to facilitate trainings for HCWs.	Lobby for financial support. Develop and distribute AHD posters
	Inadequate testing devices & tools for ART site screening;	Lobby for additional PIMA machines, Centrifuges, Automated pipettes. Refurbishment of testing room.
6. Access to knowledge and information	Inadequate AHD trained providers Staff turnovers	Facilitation of AHD trainings for additional providers. Use other platforms for learning e.g. report handovers presentations, Ground ward rounds. Promote on-jobs trainings
7. Critical incidents	COVID-19 pandemic: disruption of AHD screening services due shortage of staff and restrictions	Train lay carders to beef up the screening and testing.

**Table 4:** Summary of facilitators

**1. Formally appointed internal implementation leaders**	Skilled implementation leaders (coordinator, or team leader), with the responsibility to lead the implementation AHD package are available.	Two HIV programs Coordinators, trained in AHD Trainer of Trainers (TOT).
**2. Relative advantage**	Stakeholders see the advantage of implementing the innovation compared to an alternative solution or keeping things the same.	Respondents aware of the intervention benefits like improved diagnosis & proper management of OIs
**3. Partnerships & connections**	The organization is well networked with external organizations e.g. NGOs, MoH etc.	Lighthouse organization is supporting both ART and AHD services with their technical staff. MoH supportive supervisions and AHD review meetings
**4. External policy and incentives**	External policy documents supporting the implementation of the AHD screening package.	Guidelines for AHD management package integrated in the Malawi HIV Clinical Management guideline. Laboratory SOPs for CD4, Urine–LAM, Serum CrAg available.

## Abbreviations

ART: Antiretroviral Therapy
AHD: Advanced HIV Disease
CFIR: Consolidated Framework for Implementation Research
CM: Cryptococcal Meningitis
COMREC: College of Medicine Research Ethics
CrAg: Cryptococcol Antigen
DHSS: Director of Health and Social Services
EGPAF: Elizabeth Glaser Pediatric Aids Foundation
HCWs: Health Care Workers
HIV: Human Immunodeficiency Virus
IRIS: Immune Reconstitution Inflammatory Syndrome
MHIRST: Malawi HIV Implementation Research Scientist Training program
MoH: Ministry of Health
MPHIA: Malawi Population based HIV Impact Assessment
OIs: Opportunistic Infections
PEPFAR: President's Emergency Plan For AIDS Relief
PLHIV: People Living With HIV
SSA: Sub-Saharan Africa
TAT: Turn Around Time
TB: Tuberculosis
UNAIDS: United Nations Programme on HIV/AIDS
WHO: World Health Organization
